# The plant-sucking insect selects assembly of the gut microbiota from environment to enhance host reproduction

**DOI:** 10.1038/s41522-024-00539-z

**Published:** 2024-07-30

**Authors:** Hong-Wei Shan, Xie-Jiang Xia, Yi-Lu Feng, Wei Wu, Hong-Jie Li, Zong-Tao Sun, Jun-Min Li, Jian-Ping Chen

**Affiliations:** State Key Laboratory for Managing Biotic and Chemical Threats to the Quality and Safety of Agro-products, Key Laboratory of Biotechnology in Plant Protection of MARA and Zhejiang Province, Institute of Plant Virology, Ningbo University, Ningbo, 315211 China

**Keywords:** Symbiosis, Symbiosis

## Abstract

Plant-sucking insects have intricate associations with a diverse array of microorganisms to facilitate their adaptation to specific ecological niches. The midgut of phytophagous true bugs is generally structured into four distinct compartments to accommodate their microbiota. Nevertheless, there is limited understanding regarding the origins of these gut microbiomes, the mechanisms behind microbial community assembly, and the interactions between gut microbiomes and their insect hosts. In this study, we conducted a comprehensive survey of microbial communities within the midgut compartments of a bean bug *Riptortus pedestris*, soybean plant, and bulk soil across 12 distinct geographical fields in China, utilizing high-throughput sequencing of the 16 S rRNA gene. Our findings illuminated that gut microbiota of the plant-sucking insects predominantly originated from the surrounding soil environment, and plants also play a subordinate role in mediating microbial acquisition for the insects. Furthermore, our investigation suggested that the composition of the insect gut microbiome was probably shaped by host selection and/or microbe-microbe interactions at the gut compartment level, with marginal influence from soil and geographical factors. Additionally, we had unveiled a noteworthy dynamic in the acquisition of core bacterial taxa, particularly *Burkholderia*, which were initially sourced from the environment and subsequently enriched within the insect midgut compartments. This bacterial enrichment played a significant role in enhancing insect host reproduction. These findings contribute to our evolving understanding of microbiomes within the insect-plant-soil ecosystem, shedding additional light on the intricate interactions between insects and their microbiomes that underpin the ecological significance of microbial partnerships in host adaptation.

## Introduction

Insects are extensively colonized by microorganisms that play a pivotal role in bolstering their health and fitness. This is particularly pertinent for plant-sucking insects, which subsist on nutritionally deficient or imbalanced diets. In response, the symbiotic associations with beneficial microorganisms provide a supplementary source of nutrients^1–4^. Additionally, these symbiotic microorganisms engage in diverse interactions with their insect hosts, conferring a range of advantageous traits such as developmental support and increased fecundity^5^, enhanced tolerance to temperature fluctuations^6,7^, the detoxification of noxious chemicals^8,9^ and bolstered resistance to natural enemies^10,11^. The insect intestinal tract is an indispensable habitat for these symbiotic microorganisms. Generally, gut associations are transient, meaning that microorganisms can be acquired by the insect from external sources and are shed from the insect back to the environment^12,13^. Herbivorous insects are present on the plants and ingest microorganisms from the aboveground parts to incorporate in their microbiome^13,14^. Moreover, soil habitats are extremely rich in microorganisms which serve as an important microbial reservoir for insects to confer host fitness^15,16^.

Insects and their microbiomes have coevolved over millions of years, with most of their interactions proving mutually beneficial. This suggests that insects selectively attract and cultivate specific microorganisms under the pressure of natural selection, employing immune systems and providing specialized nutrients and habitats^17,18^. Concurrently, these interactions between insects and their microbiomes are also influenced by an array of environmental factors, including geographic location, host plants, and insect habitats^19–21^. Most studies on insect-symbiont interactions so far have focused on individual symbionts^22,23^. However, many insects are inhabited by a complex microbial community, the analyses of microbial community assembly and interactions between gut microbiomes and insect host remain relatively scarce.

Pentatomomorpha is one of the most diversified infraorders of true bugs (Hemiptera: Heteroptera) that are predominantly phytophagous species that extract nutrients by piercing their sucking mouthparts directly into leaves, stems, flowers, pods, and seeds^24,25^. The plant-sucking stinkbugs possess a specialized midgut that is morphologically differentiated into four distinct sections to accommodate gut microbiota^26,27^. These gut microorganisms have evolved various transmission ways, with some being vertically transmitted through mechanisms like egg surface contamination, coprophagy, or the formation and deposition of specialized symbiont-containing capsules^28–30^. Others are acquired horizontally from the environment, establishing symbiotic relationships in later life stages^31^. The transmitted journey begins with newly hatched true bug nymphs, which are initially devoid of symbiotic microorganisms and subsequently acquire symbionts by probing with their proboscises^12^. Ultimately, some beneficial microbes are selectively enriched in the specific regions (M3 or M4 section) of insect midguts^5,32^. Despite considerable interest the interaction between the stinkbug and their gut symbionts, little is known on the microbial community and the enriched process of specific taxa in the different midgut compartment niches.

This study centers around the investigation of gut microbiota in the bean bug *Riptortus pedestris*, a notorious pest of leguminous crops, and the cause of soybean “staygreen” syndromes across many regions in Asia^33,34^. The insect is extensively associated with a bacterial symbiont belonging to the genus *Burkholderia* in crypts at a posterior midgut region^35^. *Burkholderia* symbionts are characterized by environmental acquisition and provide beneficial effects on the development of the insect hosts^23,31^. Thus, the stinkbugs and their symbionts serve as ideal subjects for examining the potential sources of gut microbiota with horizontal acquisition and the determinants of partner specificity within hosts. In the study, we conducted a comprehensive survey covering a wide geographic scale of bacterial communities across plant, soil, and multiple insect midgut compartments within soybean fields. The purpose of the investigation is to unravel the intricate processes through which the insect host and their environments influence the assembly of microbiomes and co-occurrence patterns across various gut compartments in the insects. Additionally, we aim to decipher the underlying interactions that may hint at a potentially symbiotic relationship between key bacteria and host fitness.

## Results

### Bacterial communities in different gut compartments of *R. pedestris* and their surrounding environment

**Fig. 1:**
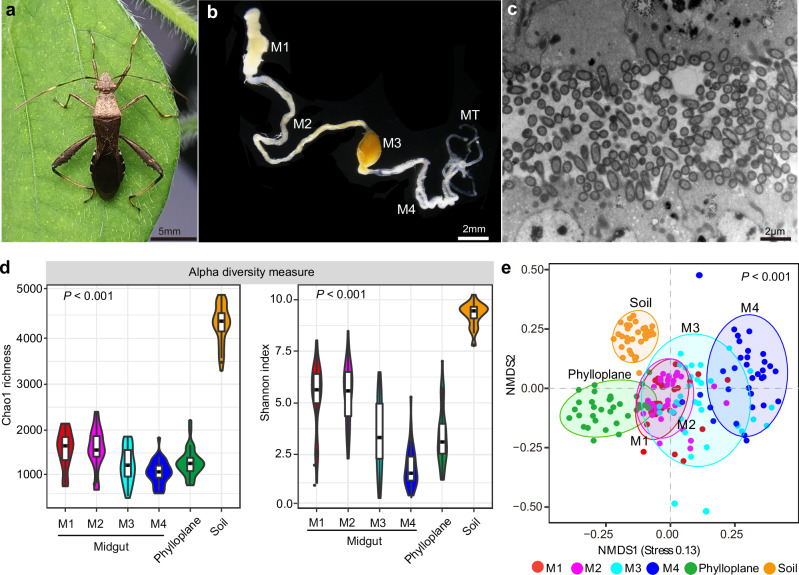
The diversity of bacterial community within insect midgut, phylloplane and soil of soybean fields. **a** A stinkbug *R.pedestris* live on a soybean leaf. **b** The insect midgut is differentiated into four sections; M1-M4, the four sections of the midgut; MT: malpighian tubes. **c** Transmission electron micrographs (TEM) of the insect midgut with a bacterial layer. **d** Chao1 and Shannon diversity metrics of the four sections of insect midgut, phylloplane and soil of soybean field collected from the insect habitat. **e** Non-metric multidimensional scaling (NMDS) of bacterial communities based on Bray-Curtis similarity. The difference of microbial communities among the different samples was calculated with PERMANOVA via “adonis” test. The insects, soybean leaves and soils were collected from 12 different geographical areas in China, and see Supplementary Table 1 for details of the sample collection information.

The midgut of the bean bug *R. pedestris*, akin to many other stinkbugs, exhibits differentiation into four morphologically distinct sections: the first midgut section (M1), which is notably voluminous; the second midgut section (M2), characterized by its elongated tubular shape; the ovoid third midgut section (M3); and the fourth midgut section (M4), hosting numerous crypts and typically colonized by a substantial bacterial population (Fig. 1a–c). We then characterized the microbiomes within these four sections of the insect midgut, as well as the neighboring plant and soil compartments, using Illumina MiSeq sequencing based on the 16 S rRNA regions. A total of 210 microbial community samples were collected from soybean fields across twelve geographical areas in China and subsequently sequenced. Rarefaction curves were generated using all the samples to calculate alpha diversity (Supplementary Fig. 1). The highest bacterial diversity was observed in the soils, followed by the moderately diverse phylloplane (leaf surface), while a clear trend of gradually decreasing alpha diversity was evident from M1 to M4 sections within the insect midgut (Fig. 1d). Distinct bacterial beta diversity was observed across four insect midgut sections, phylloplane, and soil (Fig. 1e, adonis, *R*^*2*^ = 0.24, *p* < 0.001). Non-metric multidimensional scaling analysis (NMDS) based on Bray-Curtis distances among communities revealed the presence of five distinct sample clusters, corresponding to the sample type. Notably, M1 and M2 of the midgut nearly overlapped in the NMDS plot (Fig. 1e). Furthermore, the first three midgut sections displayed partial overlap with the phylloplane and were closer in composition to the soil compartments than the M4 section of the midgut (Fig. 1e).

### The potential sources and selection processes of insect intestinal microbiome

**Fig. 2:**
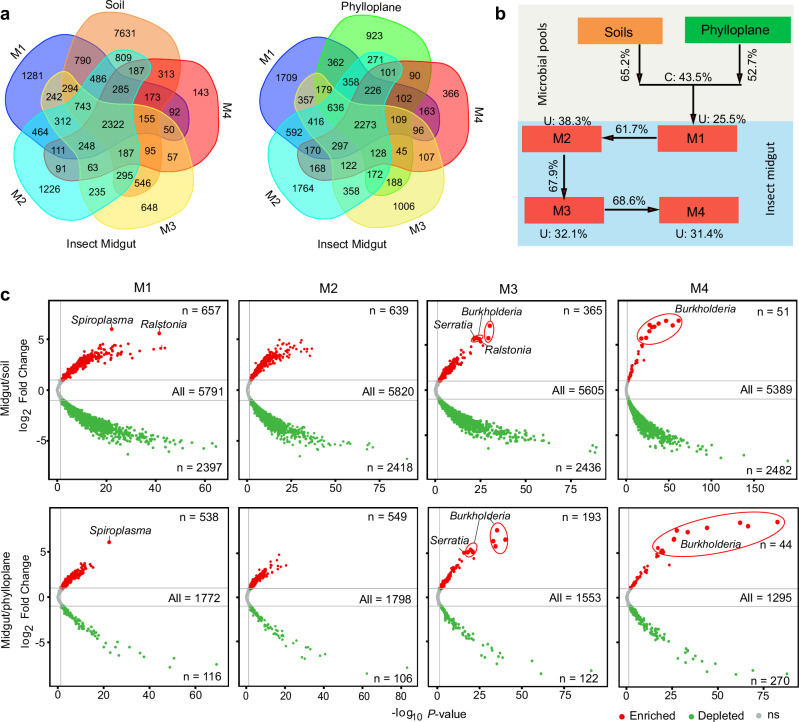
The insect intestinal bacterial communities are mainly derived from soil and phylloplane, and gradually enriched and filtered by different gut compartment niches. **a** Venn diagrams showing the shared operational taxonomic units (OTUs) in different insect midgut compartment niches within soil/phylloplane. **b** Source model of microbiome show the potential sources of insect midgut-associated bacterial communities based on samples collected in soybean fields. U, unknown source. **c** The volcano plot illustrating the enrichment and depletion patterns of the gut-associated bacterial microbiomes in each compartment niche compared with soil and phylloplane. Each point represents a single OTU, each red point represents an individual enriched OTU and a green point represents an individual depleted OTU (log2 FC > 1; *p* < 0.05). The extremely enriched OTUs (log2 FC > 5) are labeled with their genus. The position along the y-axis represents the fold-change (FC) in abundance compared with soil and phylloplane, and the x-axis represents the *p*-values with *t*-test.

To further elucidate the potential origins and processes of host selection for the intestinal microbiota, we conducted source-tracking analysis to pinpoint the bacterial communities observed within insect midguts, as well as the neighboring plant and soil compartment niches. Notably, the insect guts shared the majority of Operational Taxonomic Units (OTUs) with both the soils and plant leaves. Furthermore, there was a discernible decline in the common OTUs from M1 to M4 sections of the insect midgut (Fig. 2a). The Source Model of Gut Microbiome (SMGM) provided insights into the contributions of different sources to the insect gut-associated bacterial communities. It revealed that these communities originated from two primary reservoirs: bulk soils and plant leaves, which together contribute ~75% of the microbial composition. Specifically, soils emerged as the dominant microbial source with known source values exceeding 65%, while plant leaves played a secondary role with known source values exceeding 52% (Fig. 2b). Additionally, the bacterial communities appeared to undergo gradual filtration as they transitioned through the four distinct midgut compartments (Fig. 2b).

The differential abundance analysis revealed notable findings: within the insect midgut sections, 11.3% of OTUs (657 out of 5791 OTUs) in M1, 11.0% of OTUs (639 out of 5820 OTUs) in M2, 6.5% of OTUs (365 out of 5605 OTUs) in M3, and 0.9% of OTUs (51 out of 5389 OTUs) in M4 were significantly enriched when compared to the soils (Fig. 2c). Additionally, some bacteria were specifically enriched in the insect different midgut compartments. For instance, in the M1 midgut sections, the most abundant OTUs (with log2 fold change > 5) belonged to the genera *Spiroplasma* and *Ralstonia*. In the M3 midgut section, OTUs from the genera *Burkholderia*, *Serratia*, and *Ralstonia* were mostly enriched. Interestingly, all nine OTUs significantly enriched in the M4 sections belonged to the genus *Burkholderia* (Fig. 2c). Comparatively, when compared to plant leaves, the insect hosts exhibited a similar pattern of enrichment in bacterial communities across the various midgut compartments (Fig. 2c).

### Spatial dynamics of microbial co‑occurrence networks in insect midgut

**Fig. 3:**
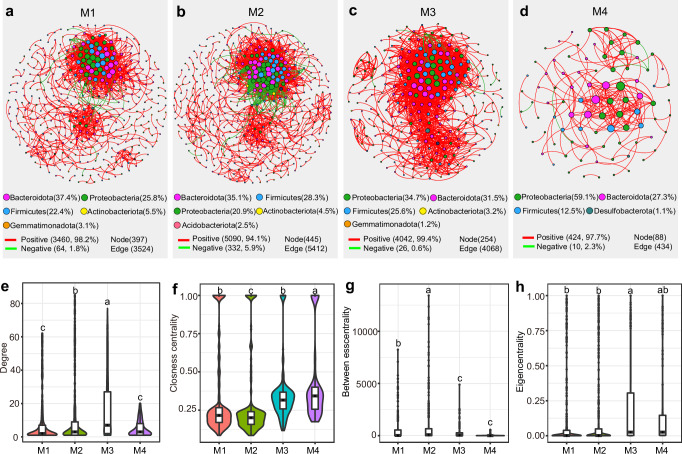
Spatial dynamics of microbial network in different insect midgut compartment niches. Co-occurrence network analysis of full dataset (*n* = 144) showing microbial network patterns differed among M1(**a**), M2(**b**), M3 (**c**) and M4 (**d**) sections of insect midguts. A connection indicates a strong (Spearman’s coefficient > 0.6) and significant (*p* < 0.01) correlation. The node size is proportional to the number of connections (i.e., the degree). The edge color represents the Spearman correlation, where green edges represent negative interactions and red edges represent positive interactions. Comparison of node-level topological features including the degree (**e**), closeness centrality (**f**), betweenness centrality (**g**) and eigenvector centrality (**h**) among the four midgut sections. Different letters indicate significance at the *p* < 0.05 level as determined by One-way ANOVA following LSD test for multiple comparisons.

We conducted a comprehensive co-occurrence network analysis to investigate how structural differentiation within the host intestine impacts microbial interactions. Our findings revealed distinct shifts in co-occurrence patterns across the four compartment niches (M1-M4) of the insect midgut (Fig. 3). Specifically, the phyla Bacteroidota, Proteobacteria, and Firmicutes emerged as dominant bacteria, collectively occupying key positions, accounting for over 85% of the network in all four midgut sections (Fig. 3a–d). Notably, the prominence of these three phyla varied, with Proteobacteria gradually gaining dominance from section M1 to M4 (Fig. 3a–d). The most notable difference lay in the network of the M4 section, which displayed fewer correlations and exhibited a looser network structure compared to the other three midgut sections. In contrast, the networks in sections M1, M2, and M3 showcased more complex structures, characterized by a higher node count, greater connectivity, and increased network density compared to the M4 section (Fig. 3a–d). Furthermore, we examined specific node-level topological features within the four networks, revealing significant differences in degree, closeness centrality, betweenness centrality, and eigenvector centrality values across the sections (Fig. 3e–h). The intricate nature of these networks underscored the substantial influence of host intestinal structural differentiation on the assembly of microbial communities.

### Environmental factors affect insect gut bacterial communities

**Fig. 4:**
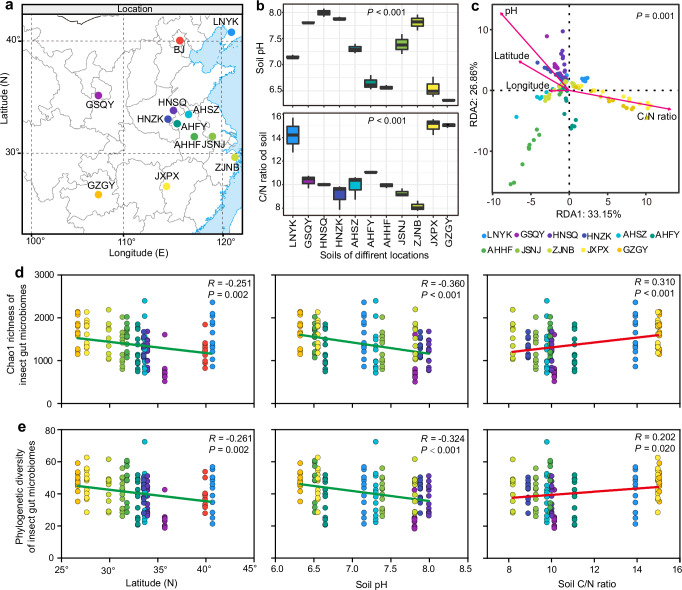
Effects of geography and soil chemical properties on insect gut bacterial diversity. **a** The sampling sites. For details of the sample collection information, see Supplementary Table 1. **b** The pH and organic carbon/total nitrogen contents (C/N) of soils from different sampling sites. The organic carbon contents and total nitrogen contents is presented in Supplementary Fig. S2. **c** Distance-based redundancy analysis (db-RDA) of the insect bacterial community with abiotic variables across different geographic areas. **d** The effect s of latitude, pH and C/N ratio of soils on the Chao1 richness of insect gut bacterial communities. **e** The effects of latitude, pH and C/N ratio of soils on the phylogenetic diversity of insect gut bacterial communities. The correlations are based on linear Pearson correlation and the significance of the correlation is at *p* < 0.05.

To explore the impact of environmental factors on insect gut microbiomes, we conducted an analysis to investigate the relationship between gut microbial diversity and the surrounding geographic and soil conditions. Our sampling sites covered a wide range of latitudes and longitudes (Fig. 4a, Supplementary Table 1), with significant variations observed in soil chemical properties, including pH, soil organic carbon (SOC), and total nitrogen (TN) content, across the geographic sites (Fig. 4b; Supplementary Fig. 2). The results of the distance-based redundancy analysis (db-RDA) highlighted that latitude, soil pH, and C/N ratios were the primary drivers of variation in the diversity and community composition of insect gut bacteria (Fig. 4c). Subsequently, we examined the correlations between insect gut bacterial alpha diversity (Chao1 richness and phylogenetic diversity) and environmental factors using linear Pearson correlation coefficients. Further analysis revealed significant negative correlations between diversity indices and latitude (Fig. 4d, e, *p* = 0.002 for both Chao1 richness and phylogenetic diversity). Similarly, soil pH exhibited significant negative correlations with the two diversity indices (Fig. 4d, e, *p* < 0.001 for both Chao1 richness and phylogenetic diversity). In contrast, the soil C/N ratio demonstrated significant positive correlations with the two diversity indices (Fig. 4d, e, *p* < 0.001 for Chao1 richness and *p* = 0.020 for phylogenetic diversity). Notably, other environmental factors, such as longitude, did not show significant associations with microbial diversity in our study (Supplementary Fig. 3, *p* = 0.062 for Chao1 richness and *p* = 0.107 for phylogenetic diversity).

### Taxonomic composition of bacterial communities in four midgut sections of different geographic insect populations

**Fig. 5:**
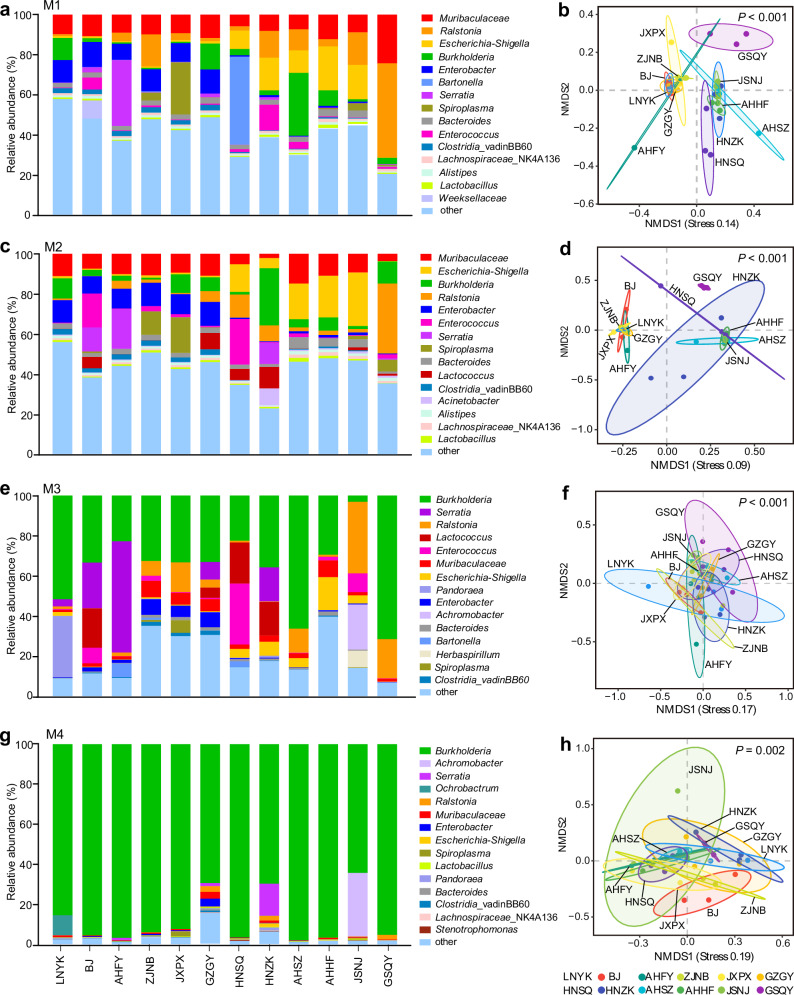
Taxonomic composition of bacterial micribiomes in four midgut sections of different geographic insect populations. **a** Relative abundance of bacterial 16 S rRNA genes in midgut M1section at the genus level from 12 insect populations. **b** Non-metric multidimensional scaling (NMDS) of bacterial communities in midgut M1section of 12 insect populations. Relative abundance (**c**) and NMDS (**d**) of bacterial communities in midgut M2 section of 12 insect populations. Relative abundance (**e**) and NMDS (**f**) of bacterial communities in midgut M3 section of 12 insect populations. Relative abundance (**g**) and NMDS (**h**) of bacterial communities in midgut M4 section of 12 insect populations. The difference of microbial communities among the insect populations was calculated with PERMANOVA via “adonis” test.

Taxonomic analysis unveiled distinct variations in microbial communities across the four insect midgut compartments, which were influenced by the geographic origin of the insect populations (Fig. 5). Specifically, the M1 and M2 gut compartments exhibited a diverse community of likely transient bacterial taxa, including genera like *Muribaculaceae*, *Ralstonia*, *Escherrichia*-*Shigella*, and *Enterobacter* (Fig. 5a, c). These bacterial communities in the two midgut sections appeared similar within each insect population, yet NMDS analysis highlighted divergence at the OTU level, resulting in the division of the twelve insect populations into two distinct sample clusters (Fig. 5b, d). The left cluster, which partially overlapped with six insect populations, showed a higher abundance of the bacterial genus *Enterobacter* and generally exhibited higher Chao1 diversity compared to the right cluster comprising the other six populations (Fig. 5a–d and Supplementary Fig. 4a, b). Additionally, the PERMANOVA test based on Bray-Curtis distance measures revealed that the bacterial community structures of the two midgut sections were significantly different among the twelve geographical insect populations (Fig. 5b and d, adonis, *R*² = 0.56, *p* < 0.001 for M1 and *R*² = 0.52, *p* < 0.001 for M2). Conversely, microbial communities in the M3 and M4 sections of the insect populations tended to display a more uniform pattern (Fig. 5e–h). Notably, the genus *Burkholderia* became increasingly abundant in the M3 sections, eventually emerging as a core taxon that dominated the M4 compartment niches, accounting for over 90% of the microbial composition in most insect populations. This dominance of *Burkholderia* effectively excluded other competing taxa in the M4 sections (Fig. 5g, h). Despite the microbial communities within the midgut M3 and M4 sections showing a relatively consistent pattern, there were still some variations across the geographical insect populations (Fig. 5f and h, adonis, *R*^*2*^ = 0.49, *p* < 0.001 for M3 and *R*^*2*^ = 0.47, *p* = 0.002 for M4). Simultaneously, alpha-diversity richness exhibited a declining trend in these two midgut sections (Supplementary Fig. 4c-d).

### The gut-associated bacteria facilitate insect host reproduction

**Fig. 6:**
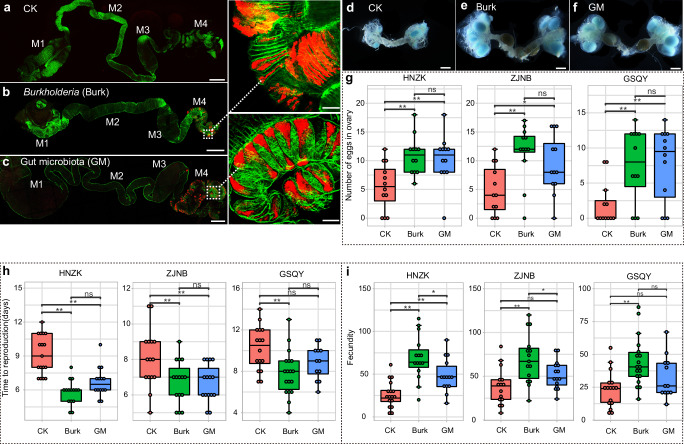
Gut microbiota enhances insect reproduction. Fluorescent in situ hybridization shows the bacteria in the midgut of *R. pedestris* that are inoculated with sterile water (**a**, CK), core bacteria *Bulkhoderia* (**b**, Burk) and homogenates of midgut of adults from their original populations (**c**, GM). Red and green signals indicate bacteria 16 S rRNA DNA and F-actin of insect midgut, respectively. M1, M2, M3 and M4 are the first to fourth section of insect midgut; Scale bars: 1 mm for the entire field of midgut and 100 μm for the magnification in M4. **d**–**f** The ovary of insect adults that are emerged in the 5th day. Scale bars: 1 mm; The developing eggs and mature eggs are observed in the ovarioles and oviduct, respectively. **g** The number of eggs in the ovary of the 5th-day adults that are inoculated with different bacteria. HNZK, ZJNB and GSQY are three insect populations collected from Zhoukou, Henan Province (33.54°N, 114.61°E), Ningbo, Zhejiang Province (29.90°N, 121.84°E) and Qingyang, Gansu Province (35.73°N,107.65°E), respectively. **h** Time to reproduction after the emergence of adults in three insect populations. **i** Number of eggs per female produced in 15 days after the emergence in the three insect populations. The asterisks indicate significant differences (*p < 0.05; **p < 0.01; ns, no significant).

The aforementioned findings revealed that the genus *Burkholderia* stood out as the core bacterial taxon consistently present in the insect midguts of all tested populations. To assess the impact of the bacteria on host reproduction fitness, we conducted experiments involving three different insect populations, each inoculated with their respective original gut bacterial strains. Initially, we isolated three distinct *Burkholderia* strains belonging to the “stinkbug-associated beneficial and environmental (SBE)” group, originating from the ZJNB, HNZK, and GSQY insect populations respectively (Supplementary Fig. 5). Subsequently, we inoculated the insects with these isolated *Burkholderia* strains or homogenates of midgut contents obtained from adults of their original populations respectively, thereby establishing a symbiotic relationship with the core bacteria *Burkholderia*, primarily within the crypts of their midgut M4 sections (Fig. 6a–c). Concurrently, the insects also colonized other gut bacteria in the four different midgut sections through the midgut homogenate inoculation (Supplementary Fig. 6). Following gut bacteria inoculation, the insects exhibited increased body weight in both female and male adults (Supplementary Fig. 7). Moreover, the presence of gut bacteria promoted the development of insect ovaries (Fig. 6d–f), leading to a higher number of eggs within the ovaries and an accelerated maturation of these eggs (Fig. 6g and Supplementary Fig. 8). Subsequently, the symbiotic insects initiated reproduction earlier and demonstrated improved egg production (Fig. 6h, i).

## Discussion

Understanding the potential sources of insect-associated microbiomes can provide critical information on the interactions among insects, microbes and their environment^36–38^. Many plant-sucking insects like aphids, whiteflies and leafhoppers harbor endosymbiotic bacteria transmitted vertically from females to eggs. However, the stinkbugs *R. pedestris* is an exception that are associated with gut bacteria. In most cases, insect gut bacteria are horizontally transmitted, meaning that microorganisms can be acquired by the insect from external sources and are closely linked to their habitat environment. Given that phytophagous insects primarily feed on plants, which host a diverse array of microbes, it is plausible that these plants play a role in shaping the microbial composition of the insect gut^39,40^. However, previous studies have indicated that certain true bugs begin life as symbiont-free at the newly hatched stage, subsequently acquiring the genus *Burkholderia* into their midguts from the soil or rhizosphere during later developmental stages^31,41^. Considering that *R. pedestris* frequently feeds on legume crops (Fig. 1a), our survey of the microbiomes in soybean fields’ plants and soils suggests that these environments could potentially serve as reservoirs for the insect gut microbiota.

Our data revealed significant differences in microbial diversity among insect guts, plants, and soils, with soils harboring the most abundant microorganisms in the bean field ecosystem (Fig. 1d). Furthermore, the microbiome communities within insect guts shared a greater number of OTUs with soils than with plant communities (Fig. 2a). Source tracking analysis further supported that soil microbiomes could make a more substantial contribution to the composition of insect gut microbial communities (Fig. 2b). Nonetheless, it’s worth noting that plants typically harbor a portion of the soil microbiota, with microbial communities settling in both the roots and above-ground parts during their growth and development^19,38^. Concurrently, herbivorous insects are often found on plants, potentially facilitating the transfer of soil microbiota into the microbiota of insects through their interactions with plants^42,43^. Our data also indicated that the bacterial communities within insect midguts partially overlapped with those found in the phylloplane (Fig. 2a, b), suggesting that plants might indeed play a role in the acquisition of bacteria by the stinkbug insects.

In agricultural ecosystems, the microbial communities are often influenced by multiple environmental factors due to various climates and complex biological networks^44,45^. For example, previous studies indicated the root and soil microbiomes are linked with soil properties and geographic factor^46–48^. Consequently, we hypothesized that the insect gut microbiotas, originating from environmental sources, are also shaped by such abiotic factors. Our findings demonstrated that soil properties, including pH and C/N ratio, were significantly associated with the diversity of the gut microbiome in *R. pedestris* (Fig. 4c–e). This suggests that soil habitats serve as the primary sources of insect microbiome and indirectly exert their influence on the microbial communities of these terrestrial creatures. Additionally, geographical factors, primarily latitudes, also had notable effects on bacterial diversity among insects collected from different sites (Fig. 4c). Specifically, *R. pedestris* hosted gut bacterial communities with higher richness and phylogenetic diversity at lower latitudes (Fig. 4c–e), implying that higher temperatures may accelerate the boom of bacterial communities in insect guts. This pattern aligns with findings in other insects, such as honeybees, flies, and plant hoppers, where bacterial communities were similarly influenced by geographical factors^20,21,49^.

In addition to facing abiotic stresses from their habitat environment, the assembly of the insect gut microbiome is also heavily influenced by host factors^50–52^. In stinkbugs, the midgut is subdivided into a series of structurally distinct regions, each harboring different microbial communities^26,27,53^. In our study, we observed that the insect gut-associated bacterial communities were derived from soils and plants, and were subsequently filtered and enriched in different gut compartment niches (M1 to M4 midgut sections) (Fig. 2b, c). Furthermore, we observed that the bacterial richness and network complexity reduced across the four midgut compartment niches, with the lowest bacterial diversity recorded in the end sections of the midgut (Fig. 3). These findings collectively suggest that insects may recruit microbes that are well-suited to specific niches within their gut compartments, and this assembly process gradually enriches certain taxa while filtering out others. The selective inoculation of environmental bacteria is likely shaped by both host-driven selection and microbe-microbe interactions. The insect gut has evolved into distinct compartments that may exhibit varying microenvironments, including pH and oxygen levels, capable of supporting diverse microbial communities^54^. Moreover, insects possess a specialized gut structure featuring narrow channels known as “constricted regions”, which display antimicrobial properties and serve a role in filtering microbes, thereby selecting specific microbial partners for distinct gut segments ^26,55,56^. Conversely, the coexisting microbes engage in a variety of interactions. These interactions may involve competition, where certain microbes with higher competitiveness dominate the niche^57^, or cooperation, which enhances the benefits derived from symbiotic partners, thus maintaining a stable gut microbiota^58^.

Our study demonstrates that both the host and geographical site significantly influence the bacterial communities within different gut compartment niches of insects, underscoring the joint impact of host-microbe interactions and environmental factors on the composition of gut microbes^51,59^. Our research uncovers significant disparities in microbial communities across various geographic populations of insects, especially noticeable within the M1 and M2 midgut sections. Conversely, microbial communities within the M4 midgut compartment of insects from geographically distant regions demonstrate a relatively consistent pattern, albeit with some variations between groups (Fig. 5). The microbial communities in the front sections of the midgut, such as M1 and M2, are likely directly influenced by environmental abiotic factors, while the M4 section, characterized by numerous crypts specialized for hosting symbiotic bacteria, appears to be also shaped by host selection and microbial-microbial interactions. Collectively, these results provide valuable insights into the dynamic relationship between hosts and microbes. This intricate process likely involves host recruitment, microbial filtration and the enrichment of specific taxa in various gut niches^55,56^^,^^60^. Meanwhile, microbes may engage in complex interactions, rapidly evolving and adapting to different gut environments^57,58^.

Mutualistic symbiosis in this context suggests that insects tend to attract and selectively favor microbial taxa that confer fitness advantages to their hosts^37,61^. The genus *Burkholderia* is frequently found in pentatomomorphan stinkbugs, particularly in insects belonging to the superfamilies Coreoidea and Lygaeoidea^35,62–64^. Our findings illuminate the journey of *Burkholderia*, originating from the environment and subsequently undergoing filtration and enrichment within different midgut compartment niches of *R. pedestris*. Ultimately, *Burkholderia* emerges as the dominant microbial taxon, accounting for up to 90% of the microbial composition in the M4 midgut section across most of the twelve tested insect populations (Fig. 2c and Fig. 5). The establishment of this intimate symbiotic relationship between insects and environmental *Burkholderia* is determined by both host intestinal structure and microbial characteristics. Specifically, stinkbugs have evolved a specialized narrow passage known as the “constricted region” between the inner cavities of the M3 and M4 midgut sections, which permits *Burkholderia* to access the M4 section while blocking the entry of other bacteria^58,64^. Simultaneously, the M4 section undergoes morphological modifications, transforming into substantial sac-like structures known as crypts and developing an extensive tracheal network that envelope the M4 section, ensuring sufficient oxygen for respiration to accommodate bacterial symbionts^26^^,^^65^. In addition, the distinct lipopolysaccharide O-antigen of the symbiotic *Burkholderia* is pivotal in initiating symbiotic associations with the host. Subsequently, the symbiont bolsters host immunity by augmenting antimicrobial activity, thereby inhibiting other microbes^66,67^. Moreover, specific antigens, flagella-mediated swimming motility, and polyester synthesis by these bacteria further enhance their adaptation to the symbiotic conditions within the insect intestine^66,68,69^.

Symbiotic microorganisms have been demonstrated to influence host reproduction in various insect groups, either by manipulating host sex ratios or by enhancing host fecundity^70–73^. Our findings indicate that gut microbiota can increase egg production in *R. pedestris*, with *Burkholderia* emerging as a key bacterial taxon exerting a significant influence over the host reproduction (Fig. 6, Supplementary Fig. 5 and Supplementary Fig. 6). Furthermore, the prevalence of *Burkholderia* suggests that the regulation of host reproduction by the gut symbionts is a common phenomenon in the natural insect populations (Fig. 6 and Supplementary Fig. 5). Symbiotic microorganisms can impact insect reproduction through various mechanisms. Numerous studies have shown that symbionts can facilitate insect development and reproduction by providing nutrients such as amino acids and B vitamins to the host^73–77^. Given that most stinkbugs are phytophagous insects that feed on nutritionally poor or unbalanced diets such as phloem sap or xylem sap, they rely on a diverse array of symbiotic microorganisms to fulfill their nutritional requirements, ultimately affecting host fitness^2,5,32,78,79^. For example, *Burkholderia* symbionts provide hosts with the essential amino acids and cofactors in sap-sucking scale insects^80^. In a similar vein, transcriptomic data has indicated that symbiotic *Burkholderia* can produce all essential amino acids and B vitamins that are scarce in the stinkbugs’ diet, highlighting their potential role in providing nutrition to the insects^81^. Moreover, the colonization of *Burkholderia* in the host insect gut can stimulate the biosynthesis of the heteroptera-specific juvenile hormone III bisepoxide, which regulates the production of hemolymph storage proteins^82,83^. These physiological changes may subsequently lead to corresponding alterations in host reproductive processes, including ovarian development and egg maturation, thereby modulating insect reproduction (Fig. 6d–f).

In conclusion, based on a comprehensive survey covering a wide geographic scale of bacterial communities across plant, soil, and multiple insect intestinal compartments within soybean fields, this study offers a systematic understanding of the potential sources and assembly processes of the insect gut microbiome, as well as their role in regulating host reproduction. Our results suggest that soil habitats harbor an exceptionally rich microbial community, acting as main microbial reservoir that significantly contributes to the insect gut microbiome source. Additionally, plants play an important role in mediating microbial acquisition by insects. Furthermore, our study suggests that the insect gut microbiome is probably influenced by a complex interplay of both host-microbe interactions and environmental factors, leading to its unique composition in different gut compartment niches. Notably, the core bacterial taxon *Burkholderia* gradually becomes enriched while other taxa are filtered out within the insect gut compartments due to host selection and microbe-microbe interactions, ultimately influencing insect reproduction. These findings provide valuable insights into the interconnected microbiomes within the insect-plant-soil ecosystem and the intricate dynamics of insect-microbiome interactions, ultimately contributing to host fitness and ecological adaptation.

## Methods

### Sample collection

The stinkbugs were collected from 12 diverse geographic regions where the pests were widely distributed in the soybean-producing areas across China between August and October 2020 (refer to Supplementary Table 1 for details). At each location, we simultaneously collected adult insects, soybean leaves (from the mid-upper positions of the plants), and soil samples (~5 cm in depth) from three plots within the same field. The collected insects were carefully preserved in breathable containers to maintain the insects’ survival for subsequent dissection and DNA extraction. Additionally, the harvested leaves and soil samples were preserved at -80 °C to prepare them for further experimentation.

### DNA extraction and 16 S rRNA amplicon sequencing

At each of the 12 locations, three adult insect individuals were pooled to a single biological replicate, and we established three such biological replicates per location. Prior to dissection, the insects underwent a surface-sterilization process, involving a 1 min immersion in 75% ethanol, followed by thorough rinsing with sterile water for three times. The midgut was then meticulously dissected and subjected to a triple rinse with sterile water. Subsequently, the distinct four gut sections were isolated and placed into individual tubes for DNA extraction, respectively. Concurrently, approximately 10 grams of leaf samples were collected for epiphytic DNA extraction, while 2 grams of soil samples were obtained for DNA extraction in each replicate, as previously described^16,19^. DNA extraction was carried out utilizing the DNeasy PowerSoil Kit (QIAGEN, Germany), adhering closely to the manufacturer’s provided instructions.

The amplification of the V3-V4 hypervariable regions of the 16 S rRNA genes was carried out using universal primers 343 F (5’-TACGGRAGGCAGCAG-3’) and 798 R (5’-AGGGTATCTAATCCT-3’). Following amplification, the quality of the amplicons was assessed through gel electrophoresis, followed by purification using AMPure XP beads. Subsequently, the purified amplicons underwent an additional round of PCR amplification. To ensure uniform representation, equal quantities of the purified amplicons were combined for subsequent sequencing. High-throughput sequencing was performed using the Illumina Novaseq 6000 PE250 platform to generate the libraries.

The raw sequencing data were initially provided in FASTQ format. Subsequently, paired-end reads underwent a preprocessing step utilizing the Trimmomatic software^84^. This process involved the identification and removal of ambiguous bases (N), as well as the elimination of sequences with an average quality score below 20, achieved through a sliding window trimming approach. Following this trimming process, the paired-end reads were assembled using the FLASH software^85^ with specific assembly parameters set as follows: a minimum overlapping length of 10 bases, a maximum overlapping length of 200 bases, and a maximum mismatch rate of 20%. Subsequent denoising of the sequences took place in two stages: firstly, reads characterized by ambiguity, homology, or length below 200 bases were discarded. Secondly, reads where at least 75% of bases possessed a quality score above Q20 were retained. Furthermore, any reads identified as chimera were detected and subsequently removed. These two denoising steps were executed using the QIIME software^86^. The resulting clean reads underwent further processing, which entailed the removal of primer sequences and their clustering to generate operational taxonomic units (OTUs). This clustering was executed using the Vsearch software^87^, employing a 97% similarity cutoff. Within each OTU, a representative read was selected using the QIIME package. For taxonomic classification, all representative reads were annotated through a BLAST search against the Silva database (Version 123) using the RDP classifier^88^ with a confidence threshold set at 70%. Additionally, all representative reads were subjected to annotation through a BLAST search against the Unite database.

### Physicochemical analysis of soils

The soil samples were subjected to an analysis of their chemical properties, including pH, SOC, and TN. These analyses were conducted following the methods outlined by Xiao et al. ^89^. In brief, soil pH was determined by employing a 1:2.5 ratio of soil to water, utilizing the MetropH320 instrument (Mettler-Toledo Instruments Ltd., USA). For SOC and TN quantification, the dry combustion method was employed, utilizing an elemental analyzer (Vario EL III, Germany). Each measurement was carried out in triplicate for accuracy and consistency.

### The investigation of the insect gut microbiota on host reproduction

An experimental study was conducted to investigate the influence of gut microbiota on host reproduction. We selected three distinct geographical insect populations, ie., HNZK (collected at 33.54°N, 114.61°E), ZJNB (29.90°N, 121.84°E) and GSQY (35.73°N,107.65°E), and set up three different experimental treatments. (i) Aposymbiotic: In this treatment, aposymbiotic insects were obtained by subjecting eggs to a rigorous surface sterilization process, following the protocol outlined by Salem et al.^32^. Briefly, the eggs underwent a 45 s immersion in bleach (12% NaOCl), followed by a 5 min treatment with 95% ethanol. Subsequently, they were thoroughly rinsed with sterile H_2_O. (ii) Re-infected with native microbial community: In this treatment, insects were re-infected by feeding second instar aposymbiotic nymphs with homogenates obtained from freshly dissected midguts of original adults. This approach was adapted from the work of Zheng et al.,^54^. (iii) Re-infected with core bacteria *Burkholderia*: In this treatment, insects were re-infected by feeding second instar aposymbiotic nymphs with cultured *Burkholderia* strains isolated from the original adult populations, as per the methodology described by Kikuchi et al. ^8^. To verify the success of the surface sterilization and re-infection procedures, diagnostic PCR analyses were conducted using specific primers targeting *Burkholderia*^35^.

The nymphs were raised in sterile plastic containers (8 cm in diameter, 15 cm in depth) until they reached adulthood. On the 1st day of their emergence as adults, their body weight was measured. Ovary development was assessed on the 5th day of adulthood, with a focus on counting developing eggs within the ovarioles and mature eggs within the oviduct. To record the time to reproduction, a pair of newly emerged female and male were placed together, and the day when the female initiated her first oviposition was noted. To evaluate insect fecundity, the number of eggs laid by the females was recorded for a 15 day period following their emergence.

### Fluorescence in situ hybridization

The detection of bacterial distribution within the insect midgut was achieved using Fluorescence In Situ Hybridization (FISH) with the universal probe EUB338 (5′-Cy3- GCTGCCTCCCGTAGGAGT-3′) ^90^. Insect tissues were immersed in a hybridization buffer (composed of 20 mmol/L Tris-HCl with pH 8.0, 0.9 mol/L NaCl, 0.01% sodium dodecyl sulfate, and 30% formamide) containing 50 nM of the EUB338 probes per milliliter. This incubation took place overnight. Subsequently, the samples were incubated in Actin Green 488 Ready Probes for 1 h. Following this, the samples underwent thorough washing in phosphate-buffered saline and were examined under a Leica TCS SP8 X confocal microscope.

### Transmission electron microscope

We conducted an investigation into the ultrastructure of bacteria within the midgut of *R. pedestris* using Transmission Electron Microscopy (TEM). The midgut segments were meticulously separated and initially fixed overnight in 2.5% glutaraldehyde within a 0.1 mol/L phosphate buffer at pH 7.0. Following this fixation, they were postfixed with 1% OsO4 in phosphate buffer, subjected to dehydration through a graded ethanol series (ranging from 30 to 100%) and absolute acetone. Subsequently, the samples were embedded in Spurr’s resin. Thin sections of adult abdomens and eggs were obtained by precision cutting using a LEICA EM UC7 ultramicrotome, followed by staining with uranyl acetate and alkaline lead citrate for 10 min each. Finally, these prepared sections were observed using a Hitachi Model H-7650 TEM.

## Statistical analysis

We performed a comprehensive analysis of diversity, both alpha (including Chao1 Richness, Shannon-Wiener, and phylogenetic diversity indices) and beta (utilizing non-metric multidimensional scaling (NMDS) of Bray-Curtis dissimilarity), using QIIME to assess diversity variations across the samples. To assess alpha diversity, a rarefaction curve was generated to determine adequate sequence depth using QIIME. For beta diversity analysis, the bacterial community dissimilarity among different groups of samples was evaluated through permutational analysis of variance (PERMANOVA) using the “adonis” test. The significance of correlations between alpha-diversity indices and environmental factors was evaluated using Spearman correlation analysis. To explore the relationship between environmental factors and bacterial communities, we employed distance-based redundancy analysis (db-RDA). The relative abundance of various microorganisms at the OTU level served as the “response variable,” while geographical data (latitude and longitude) and soil characteristics (pH and C/N ratio) were utilized as the “explanatory variable file.” Additionally, different sampling sites were designated as the “grouping file” for analysis. Visual representation of the enrichment and depletion patterns of gut bacterial microbiomes in each compartment niche compared to phylloplane and soils was achieved through a volcano plot analysis. Only robust (log2 FC > 1; *p* < 0.05) findings were considered statistically significant. We also constructed co-occurrence networks to explore relationships among bacterial communities in different midgut sections, utilizing the igraph package and the interactive Gephi platform^91,92^. Only robust (Spearman’s *r* > 0.6 or *r* < -0.6) and statistically significant (*p* < 0.01) correlations were retained. To further analyze the networks, we calculated four topology property parameters-degree, clustering coefficient, average path length, and density-using the Network Analyzer tool in Cytoscape. Statistical significance regarding insect fitness was assessed using ANOVA analysis, and for multiple comparisons, Fisher’s least significant difference (LSD) tests were conducted at the 0.05 significance level. This analysis was performed using SPSS 20.0 Statistics software.

### Reporting Summary

Further information on experimental design is available in the Nature Research Reporting Summary linked to this article.

### Supplementary information

Supplemental Material

nr-reporting-summary

## Data Availability

The amplicon data have been deposited into the NCBI Sequence Read Archive database (https://ncbi.nlm.nih.gov) under BioProject accession number PRJNA1061644. The 16 S rRNA sequences of the isolated bacteria in this study have been deposited in the GenBank databases (https://ncbi.nlm.nih.gov) under the accession number OR856010-OR856012.

## Code availability

The underlying code used to support the findings of this study is available upon request to the authors.
