# Supplementary material for: The plant-sucking insect selects assembly of the gut microbiota from environment to enhance host reproduction
Source: NPJ Biofilms Microbiomes. 2024 Jul 30;10:64. doi: 10.1038/s41522-024-00539-z (PMC11289440; doi:10.1038/s41522-024-00539-z)
Supplement: Supplementary file 1 — Supplemental Material [file 41522_2024_539_MOESM1_ESM.pdf]

Supplementary Information for

The plant-sucking insect selects assembly of the gut microbiota from environment to enhance host reproduction

Hong-Wei Shan[1]*, Xie-Jiang Xia[1], Yi-Lu Feng[1], Wei Wu[1], Hong-Jie Li[1], Zong-Tao Sun[1], Jun-Min Li[1], Jian-Ping Chen[1]*

[1]State Key Laboratory for Managing Biotic and Chemical Threats to the Quality and Safety of Agro-products, Key Laboratory of Biotechnology in Plant Protection of MARA and Zhejiang Province, Institute of Plant Virology, Ningbo University, Ningbo 315211, China

Correspondence: Hong-Wei Shan, Email: shanhongwei@nbu.edu.cn;

Jian-Ping Chen, Email: jianpingchen@nbu.edu.cn

This file includes：

Supplementary Tables 1

Supplementary Figures 1 to 8

Supplementary Table 1. Collection details of insects, plant leaves and soils in China. Sampling site code (ID), city, province, latitude, longitude, and date of the field collections assessed here are provided.

Site ID	City	Province	Collection date	(Latitude & Longitude)	Samples		
					Insects	Plant leaves	Soils
LNYK	Yingkou	Liaoning	09/03/2020	40.18 °N, 122.12°E	+	+	+
BJ	BeiJing	Beijing	08/06/2020	39.92 °N, 116.40 °E	+	-	-
GSQY	Qingyang	Gansu	09/22/2020	35.73 °N, 107.65 °E	+	+	+
HNSQ	Shangqiu	Henan	09/29/2020	33.93 °N, 116.45 °E	+	+	+
HNZK	Zhoukou	Henan	08/20/2020	33.54 °N, 114.61 °E	+	+	+
ANSZ	Suzhou	Anhui	10/09/2020	33.64 °N, 116.98 °E	+	+	+
AHFY	Fuyang	Anhui	10/10/2020	32.91 °N, 115.86 °E	+	+	+
AHHF	Hefei	Anhui	08/14/2020	31.79 °N, 117.31 °E	+	+	+
JSNJ	Nanjing	Jiangsu	09/27/2020	31.33 °N, 118.89 °E	+	+	+
ZJNB	Ningbo	Zhejiang	09/02/2020	29.90 °N, 121.84 °E	+	+	+
JXPX	Pingxiang	Jiangxi	09/16/2020	27.62 °N, 113.87 °E	+	+	+
GZGY	Guiyang	Guizhou	08/26/2020	26.68 °N 106.62 °E	+	+	+

Note: The insects were collected in all the twelve sites, and the plant leaves and soils were collected in eleven sites except the site BJ.

Supplementary figures

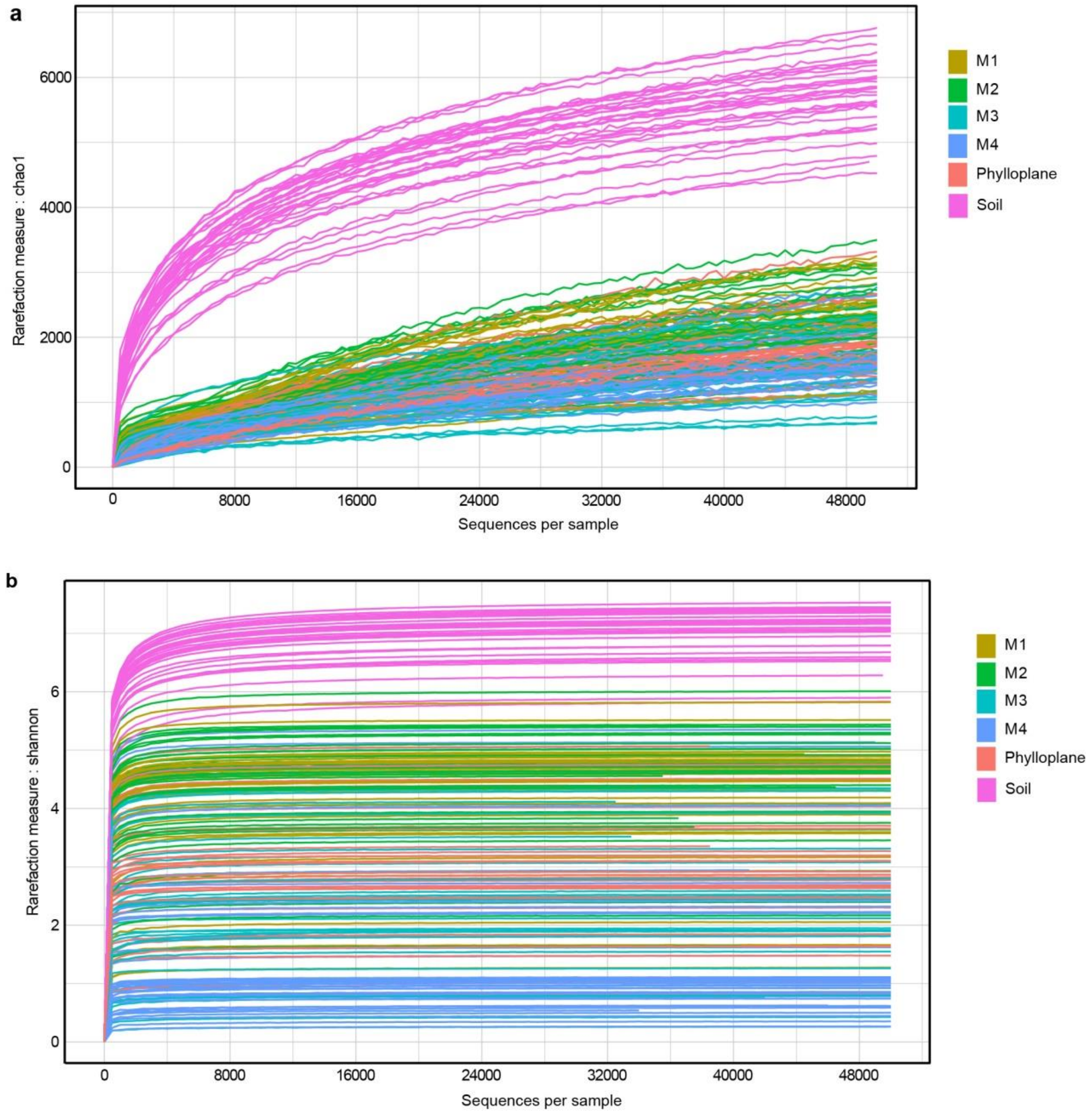

Supplementary Figure 1 Rarefaction curves showing alpha diversity for the insect four midgut sections (M1-M4), phylloplane and soil.

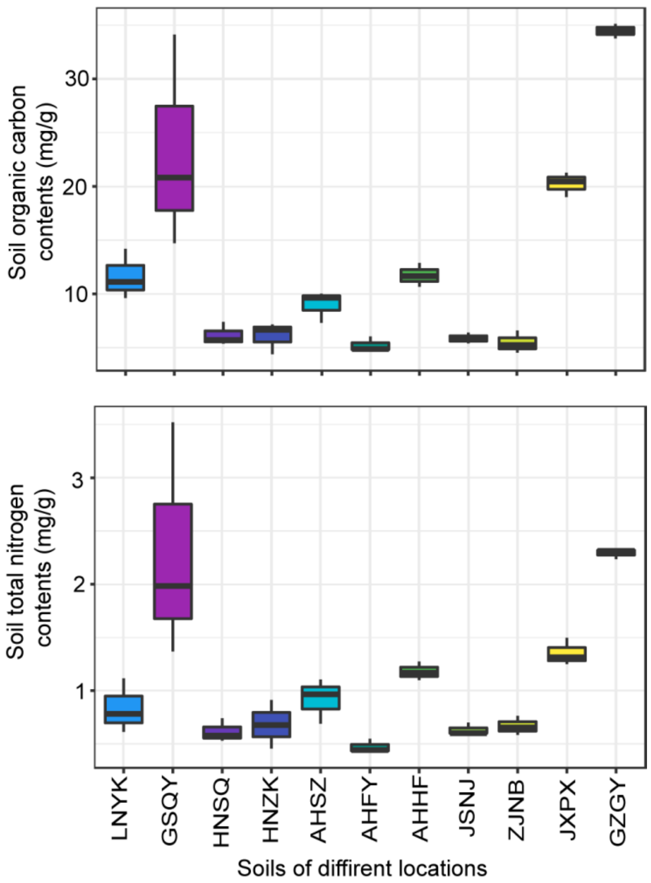

Supplementary Figure 2 Soil organic carbon contents and total nitrogen contents from different sampling sites.

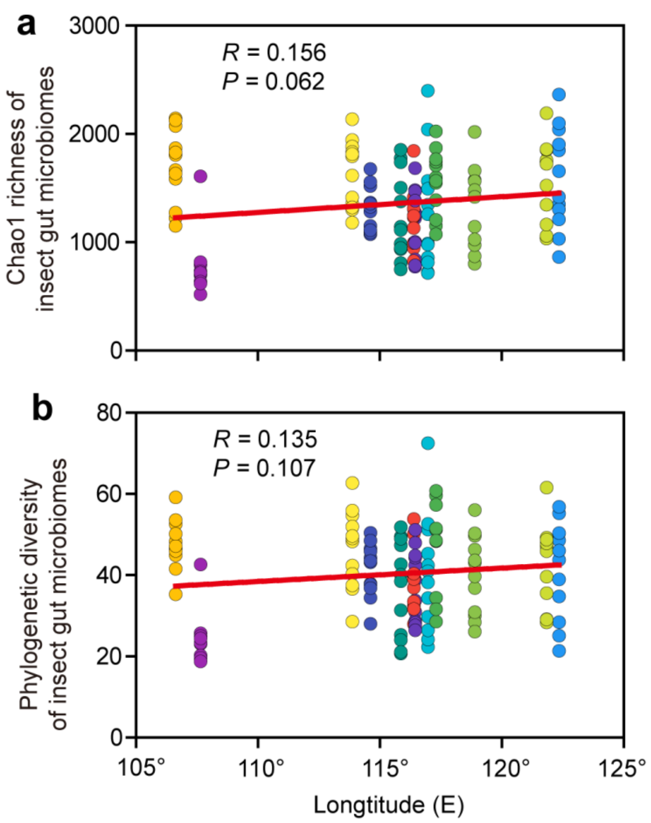

Supplementary Figure 3 The correlation of Chao1 richness (**a**) and phylogenetic diversity (**b**) of bacterial communities in insect midgut with sampling longtitude.

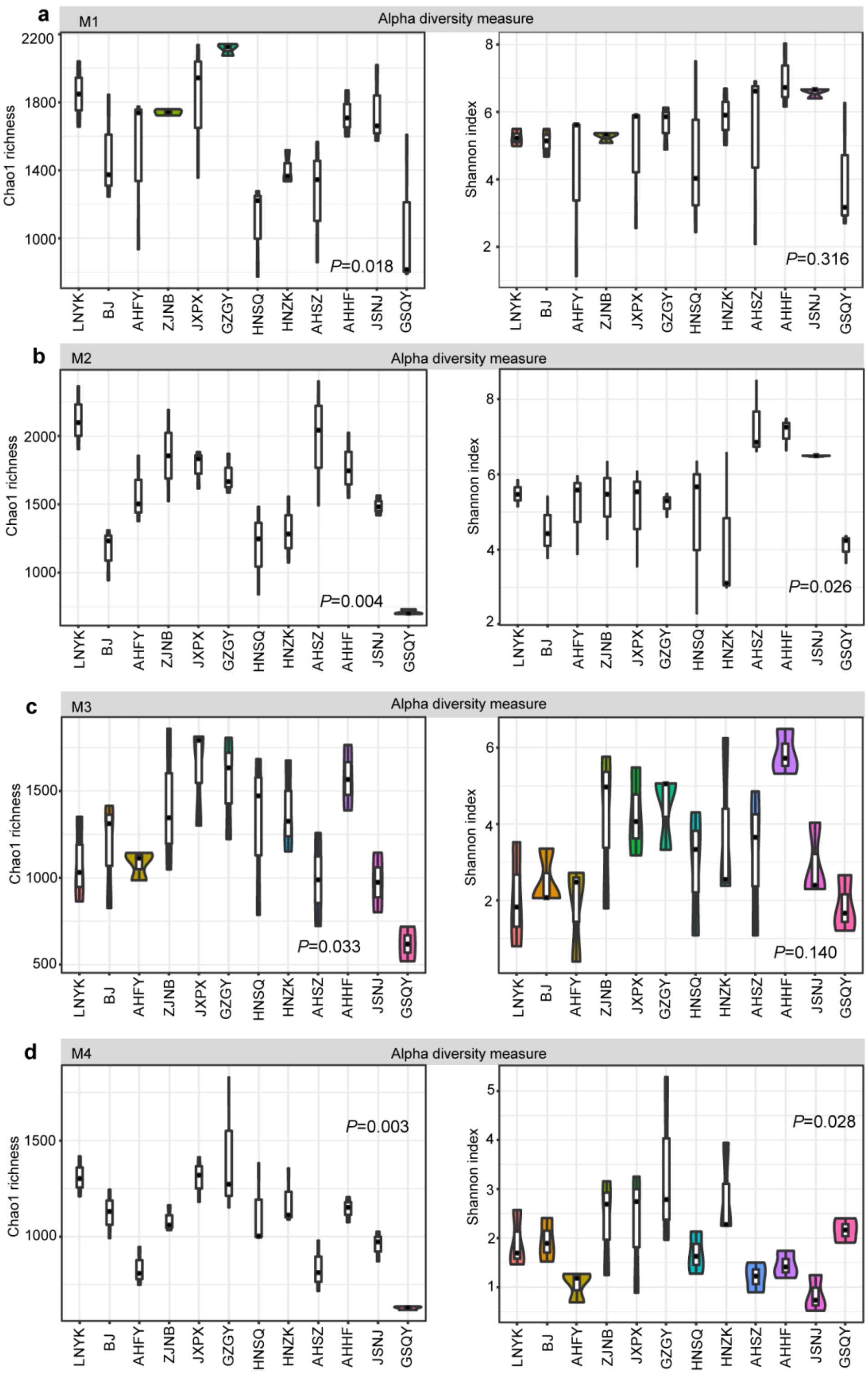

Supplementary Figure 4 The alpha diversity is estimated using Chao 1 richness and Shannon diversity index for insect midgut M1(**a**), M2(**b**), M3(**c**) and M4(**d**) compartments.

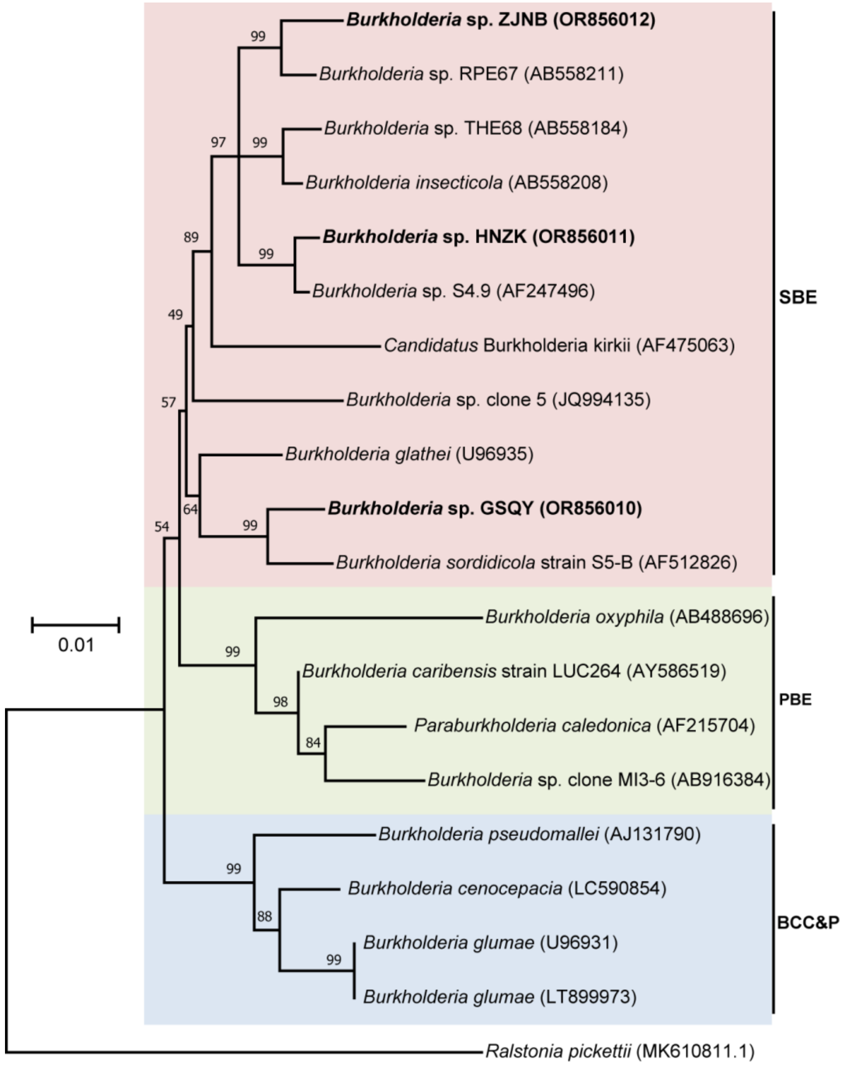

Supplementary Figure 5 Molecular phylogeny of the gut symbiotic bacteria *Burkholderia* based on 16S rRNA gene sequences. The tree displays a Neighbor-joining (NJ) phylogeny of the different *Burkholderia* groups. The newly obtained gut symbionts *Burkholderia* from three *R. pedestris* populations are highlighted in bold. Accession numbers in the GenBank are shown in brackets. Bootstrap values are depicted at the nodes. Three major *Burkholderia* clades, i.e., “stinkbug-associated beneficial and environmental (SBE)” group, “plant-associated beneficial and environmental (PBE)” group and “*B. cepacia* complex and *B. pseudomallei*”(BCC&P) group are indicated on the right.

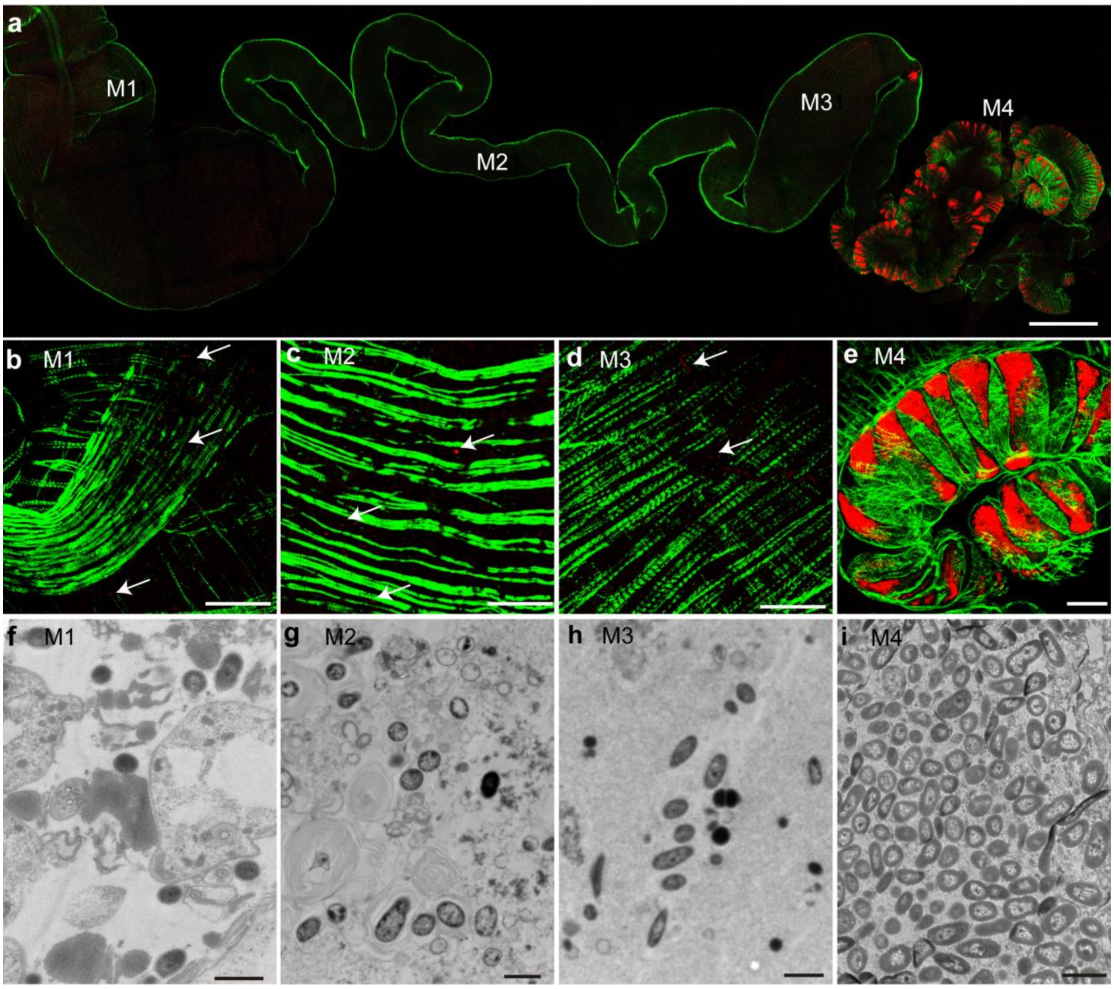

Supplementary Figure 6 The distribution of bacteria in different insect midgut sections. (**a**) The midgut was dissected from a GM insect that had been inoculated with homogenates of midgut of adults from its original population (the same midgut as that shown in Fig. 6c). M1, midgut first section; M2, midgut second section; M3, midgut third section; M4, midgut fourth section with crypts; Scale bars:1 mm. (**b-e**) High magnification of the four midgut sections. Red and green signals indicate bacteria 16S rRNA DNA and F-actin of insect midgut, respectively. White arrow, the discrete signals of bacteria in midgut M1, M2 and M3 sections; Scale bars, 100μm.

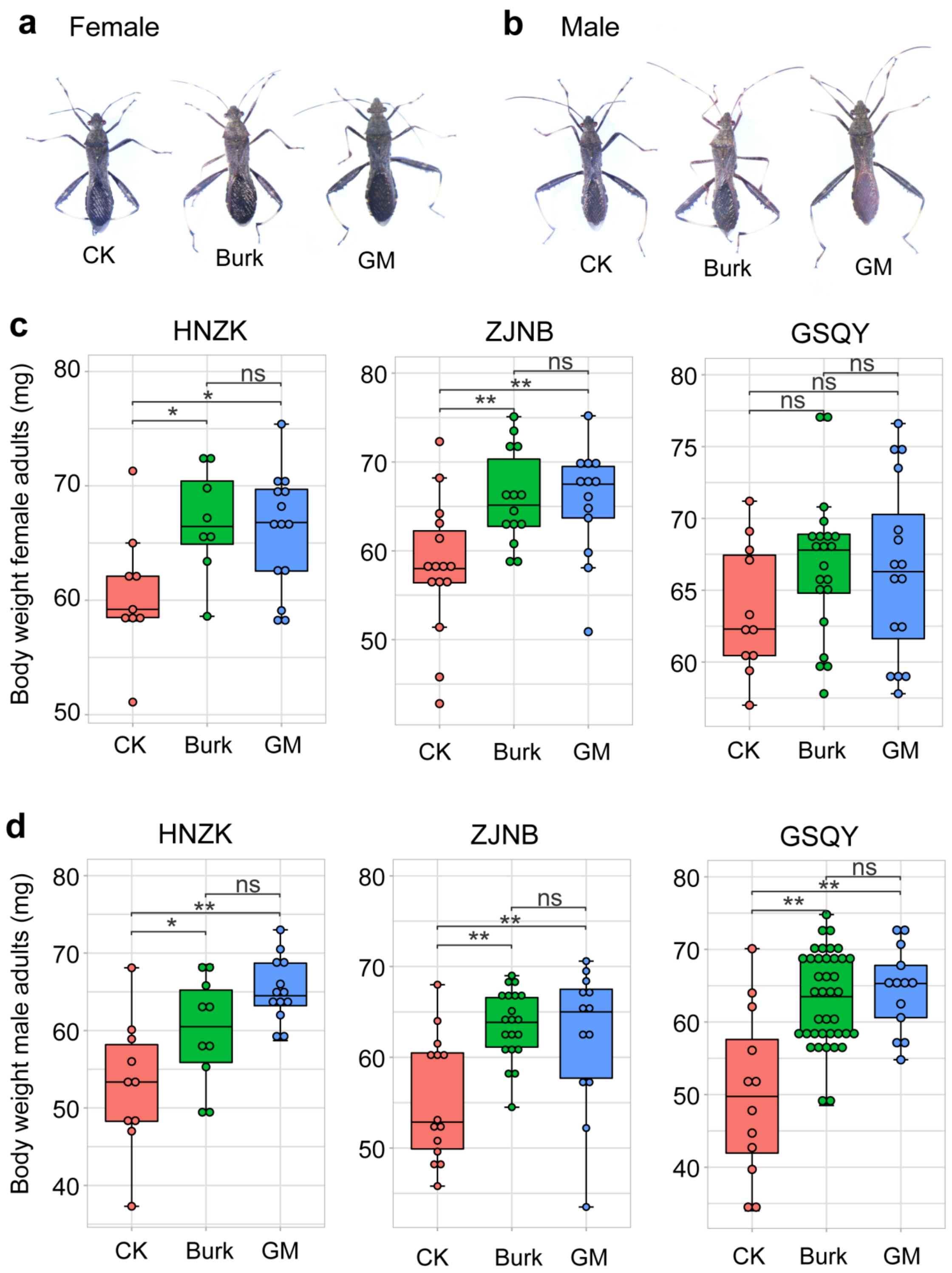

Supplementary Figure 7 Gut microbiota increases insect body weight. The first-day female (**a**) and male (**b**) adult after their emergence. The insects are inoculated with sterile water (CK), core bacteria *Bulkhoderia* (Burk) and homogenates of midgut of adults from their original populations (GM). The wet body weight of the first-day female (**c**) and male (**d**) adults of three different populations.

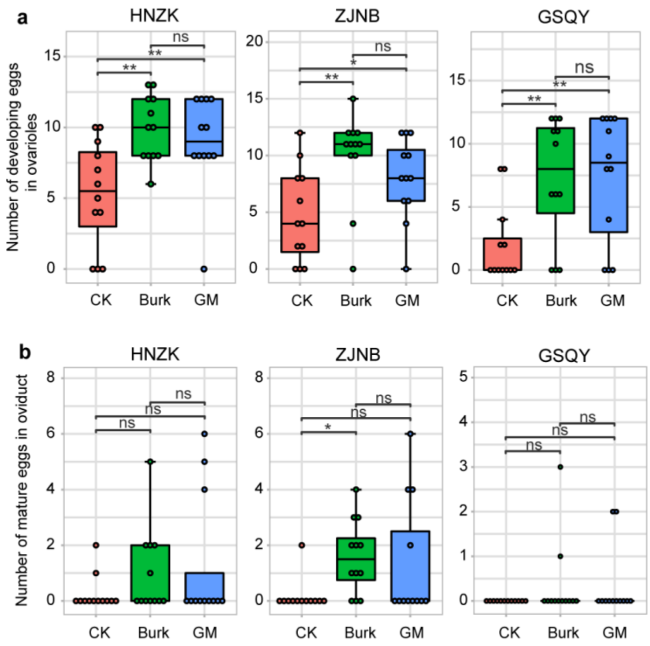

Supplementary Figure 8 The number of developing eggs (**a**) and mature eggs (**b**) in the ovarioles and oviduct of the fifth-day adults.
